# Engineered Exosomes for Tumor-Targeted Drug Delivery: A Focus on Genetic and Chemical Functionalization

**DOI:** 10.3390/pharmaceutics15010066

**Published:** 2022-12-26

**Authors:** Ali Akbari, Fereshteh Nazari-Khanamiri, Mahdi Ahmadi, Maryam Shoaran, Jafar Rezaie

**Affiliations:** 1 Solid Tumor Research Center, Cellular and Molecular Medicine Research Institute, Urmia University of Medical Sciences, Urmia 5714783734, Iran; 2 Hematology, Immune Cell Therapy, and Stem Cells Transplantation Research Center, Clinical Research Institute, Urmia University of Medical Sciences, Urmia 5714783734, Iran; 3 Drug Applied Research Center, Tabriz University of Medical Sciences, Tabriz 5665665811, Iran; 4 Pediatric Health Research Center, Tabriz University of Medical Sciences, Tabriz 5665665811, Iran

**Keywords:** exosomes, extracellular vesicles, drug delivery, nanocarriers

## Abstract

Cancer is the main cause of death worldwide. The limitations in traditional cancer therapies provoked the advance and use of several nanotechnologies for more effective and nontoxic cancer treatment. Along with synthetic nanocarriers, extracellular vesicles (EVs)-mediated drug delivery systems have aroused substantial interest. The term EVs refers to cell-derived nanovesicles, such as exosomes, with phospholipid-bound structures, participating in cell-to-cell communication. Exosomes are 30–150 nm vesicles that can transfer many biological molecules between cells. From a drug delivery standpoint, exosomes can be loaded with various therapeutic cargo, with the several advantages of low immunogenicity, high biocompatibility, transformative, and effective tumor targeting aptitude. The exosomal surface can be functionalized to improve tumor targeting ability of them. Researchers have genetically expressed or chemically linked various molecules on the surface of exosomes. Despite extensive investigation, clinical translation of exosome-based drug delivery remains challenging. In this review, we discuss various methods used to loading exosomes with therapeutic cargo. We describe examples of functionalized exosomes surface using genetic and chemical modification methods. Finally, this review attempts to provide future outlooks for exosome-based targeted drug delivery.

## 1. Introduction

**Figure 1:**
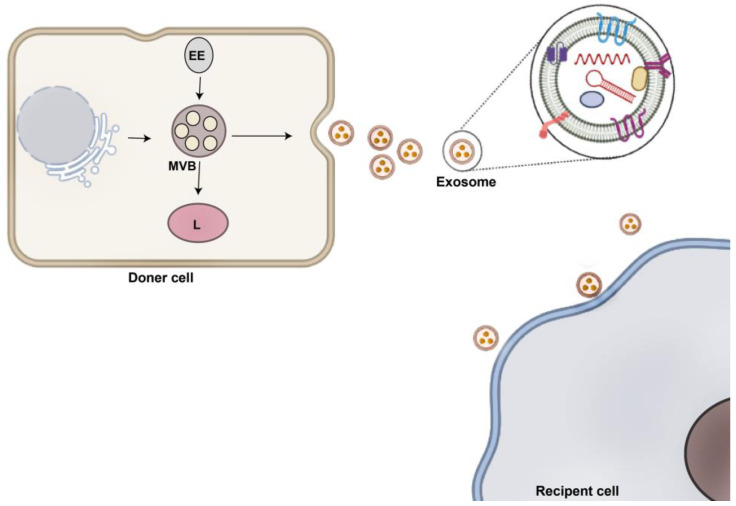
Biogenesis and secretion of exosomes. Many cells produce exosomes within multivesicular bodies (MVBs) via a complex mechanism. MVBs (a type of late endosomes) can fuse with the plasma membrane and secrete exosomes into the extracellular matrix. Now, exosomes can reach recipient cells located nearby or far from, inducing alteration in cell signaling. EE: early endosomes, L: lysosome.

Cancer is one of the most terrible illnesses worldwide. In 2022, there will be an estimated 1.9 million new cancer cases diagnosed and 609,360 cancer deaths in the United States [1]. In recent years, cancer management including detection and therapy application has been postponed because of the COVID-19 pandemic [2]. Various methods such as chemotherapy, radiation, and surgery are commonly used for malignancy treatment [3,4]. In addition, other approaches such as immunotherapy, magnetic resonance imaging, computed tomography, imaging techniques with X-ray, endoscopy, and cells/tissues morphological studies such as cytology and histopathology can assist the diagnosis and treatment of cancers [5,6,7]. However, important challenges are still ongoing in cancer therapy, which include resistance to therapies, immune escape, and metastasis. In the field of nanotechnology, nanotherapy, nanoparticles or nanocarriers at the nanoscale collection are being used to combat several diseases, which show amended drug solubility and stability, drug effectiveness, and with low side effects. In addition, the use of nanoparticles for cancer therapy is an attainment interest of researchers due to its gene silencing and tumor targeting abilities [8]. Over the decades, researchers have designed various nanocarriers for use in drug delivery systems comprising polymeric nanoparticles, micelles, liposomes, and dendrimers for increasing therapeutic efficiency. However, immunogenicity, acute hypersensitivity reaction, and biocompatibility problems are associated with these particles [9,10]. Recently, nanotechnology has progressed considerably to advance smart carriers [11]. Particularly, lipid-based nanocarriers exhibit a useful policy for drug loading that has directed to the clinical translation of some inventions [11]. With the advance of new synthetic carriers, cell derivatives particles, extracellular vesicles (EVs) have emerged as novel tools for drug delivery system [12,13]. EVs are phospholipid-bound vesicles heterogeneously deriving from cells that regulate many biological processes [14]. They carry many types of biomolecules including non-coding RNAs, proteins, signaling molecules, lipids, and DNA strands between cells, therefore trafficking to several tissues and organs and contributing to cell communication [14,15]. Various types of EVs have been characterized based on generation way and size distribution know apoptotic bodies (1000–6000 nm), microvesicles (100–1000 nm), and exosomes (30–150 nm) [16,17]. Exosomes are formed within the late endosomal compartment named multivesicular bodies, which can fuse with the plasma membrane and secrete exosomes into the extracellular matrix [18] (Figure 1). Exosomes generation and intracellular trafficking comprise many signaling processes and machinery such as tetraspanins, The endosomal sorting complexes required for transport (ESCRT) machinery, lipid rafts, Rab proteins, and SNARs [17,19,20]. Microvesicles are generated from the outward budding of the cell membrane through cytoskeleton rearrangement, the reorganization of phospholipids in the cell membrane [21,22]. Apoptotic bodies are formed by apoptotic cells through membrane blebbing, and nuclear and cytoplasmic fragmentation [23]. An increasing number of studies have shown that exosomes can carry exogenous therapeutic cargo to cells [24]. In this regard, exosomes are equivalent to liposomes because both have phospholipid membranes. However, exosomes contain a complex structure of several lipids and lumen and surface proteins; some of these surface proteins help tissue targeting [24]. The lipid bilayer of exosomes can shield their cargo from degradation. Previous studies have shown that various cargo can be loaded into exosomes with different methods. Exosomes offer several advantages because of their exceptional physicochemical characterizations [25] such as natural origin, low immunogenicity, high biocompatibility, increased stability in vivo and blood circulation time, and low toxicity [25,26,27]. Moreover, exosomes can interact with target cells through several mechanisms, allowing exosome-mediated drug delivery to a cell or tissue [28]. Exosomes surface inherits receptors from parent cells, giving confident inherent targeting capabilities. This feature is favorable to increasing the accumulation of therapeutic cargo at the target site after systemic administration, thus decreasing drug dosage for exosomal loading with low toxic side effects [29,30]. Exosome-based drug delivery can avoid the P-glycoprotein drug efflux system, which can lessen the drug resistance response in tumor cells [31]. Most importantly, exosomes are flexible regarding surface modification and engineering [32,33]. Functionalization of exosomes surface is a greatly interesting approach to increase exosomes efficacy for delivering cargo into targeted site especially in tumor studies [34]. Exosomes modification may be useful for cancer detection and imaging studies [35]. In this review, we discuss the exosome-loading methods with special focus on methods used to improve the tumor-targeting potential of exosomes.

## 2. Cargo Loading Methods

**Figure 2:**
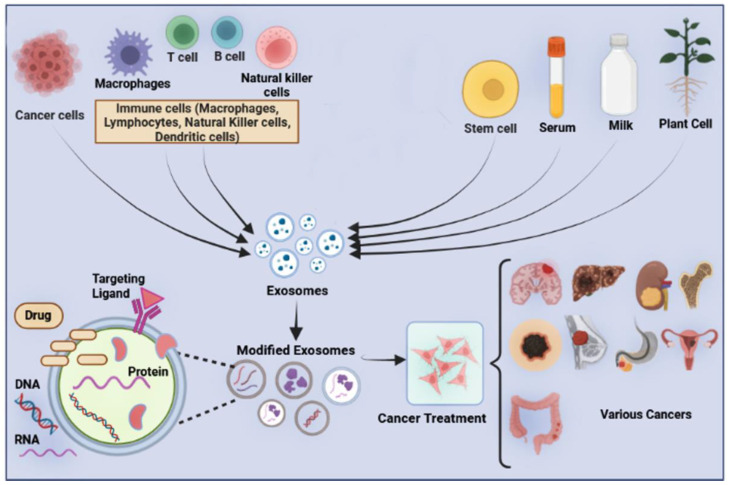
Various exosomes from different cells or body fluids are used for drug delivery system.

**Figure 3:**
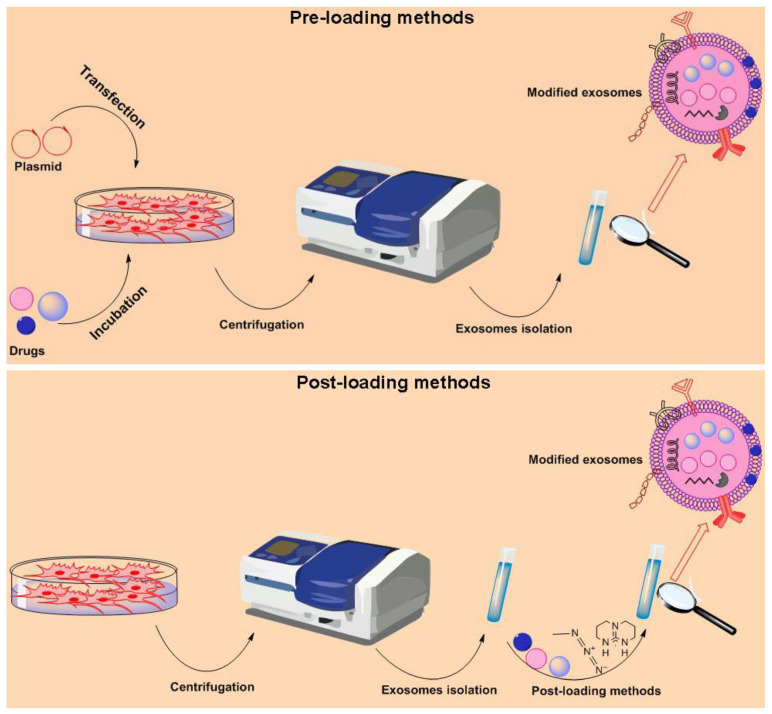
Exosomes loading methods. In general, two methods are used to load therapeutic cargo into exosomes including the pre-loading method and the post-loading method.

Exosomes from various cells are used for drug delivery systems (Figure 2). Overall, two main methods are used for cargo loading into exosomes or other EVs, known: indirect or pre-loading method and the direct or post-loading method [36] (Figure 3). In this review, we use the terms pre-loading and post-loading methods. In preloading methods, parent cells are modified and loaded with genes/cargo to produce optional exosomes. In post-loading methods, exosomes/EVs isolated from cells are directly loaded with cargo by active or passive methods. In addition, a survey on clinical trials shows that exosomes are being used for the delivery of therapeutic drugs (https://clinicaltrials.gov/ct2/home, accessed on 1 December 2022). We found that there are 6 clinical trials registered. Three clinical trials (NCT03608631, NCT03384433, and NCT05043181) were aimed at genetically modifying MSCs for producing overexpressing exosomes. Two clinical trials aimed to load curcumin into plant-derived EVs for the treatment of colon cancer Irritable Bowel Disease (NCT01294072 and NCT04879810). None of these studies is completed and results are not currently accessible. In this section, we describe loading methods used for producing exosomes.

### 2.1. Pre-Loading Method

Pre-loading methods allow continuous and easy production of cargo-loaded exosomes without conceding membrane integrity. In this method, cells were co-incubated with cargo or genetically transfected with target genes to produce cargo-loading exosomes. Therefore, co-incubation and genetic modification are two common approaches to the pre-loading method (Figure 3).

#### 2.1.1. Co-Incubation

Co-incubation is used to load different chemotherapeutic drugs, particularly hydrophobic drugs such as paclitaxel and doxorubicin [37,38,39]. In this approach, chemotherapeutic drugs are co-cultured with parental cells, then they can pass through the cell membrane and enter into MVBs where exosomes are formed and loaded. After secretion, exosomes loaded with drugs can be isolated for downstream experiments. This method is easy and straightforward on its own; however, its loading efficiency is generally low and is dependent on drug dose gradient, drug characteristics, and type of parent cell [40]. For example, doxorubicin enters into different cells with different concentrations. In other words, its loading efficacy depends on cell types [41]. To overcome this challenge, there is evidence that the pre-conditioning of cells can significantly increase loading efficacy. In this regard, low current electricity and UV irradiation may encourage the loading rate of drugs or nanoparticles into exosomes [42,43].

#### 2.1.2. Genetic Modification

In this method, therapeutic molecules including RNAs, proteins, ligands, and receptors are overexpressed in parent cells through transfection and genetic modification. Therefore, these molecules appear in the exosomes lumen or surface. Different cells such as tumor cells and stem cells were genetically modified to produce exosomes [44,45]. We focused on this method in the section below.

### 2.2. Post-loading

Through the post-loading approach, cargo are directly encapsulated cargo into isolated exosomes (Figure 3). The headmost step is selecting a safe and confident parental cell to produce mass exosomes. The second step is selecting a suitable and efficient method for inserting cargo, as loading methods may affect exosomes kinetics and structure. Active loading and passive loading are the two main methods of post-loading.

#### 2.2.1. Active Loading

Some therapeutic cargo cannot cross exosomal membranes, therefore researchers use different methods to insert cargo into exosomes by temporary induction of the permeability of exosomes membranes. As shown by Table 1, active loading methods can be mainly divided into two methods, namely physical induction and chemical induction. In physical induction methods, exosomes membrane was rapidly disrupted by external forces, including sonication, electroporation, freeze-thaw cycle, and extrusion. We summarized active loading methods in Table 1 with advantages and limitations.

#### 2.2.2. Passive Loading

In this method, isolated exosomes/EVs are co-cultured with high concentrations of drugs where drugs can diffuse into the exosomes’ lumen passively. This method is simple and easy to operate and preserves membrane integrity. A growing body of evidence indicates that passive loading is widely used for cancer studies [60,61,62]. Nonetheless, this method has limitations such as low loading efficiency and dependent on the nature of drugs (hydrophobic compounds). For example, the loading efficacy of paclitaxel and curcumin is different upon co-incubation at the same condition [63,64].

## 3. Improve Tumor-Targeting Potential of Exosomes

**Figure 4:**
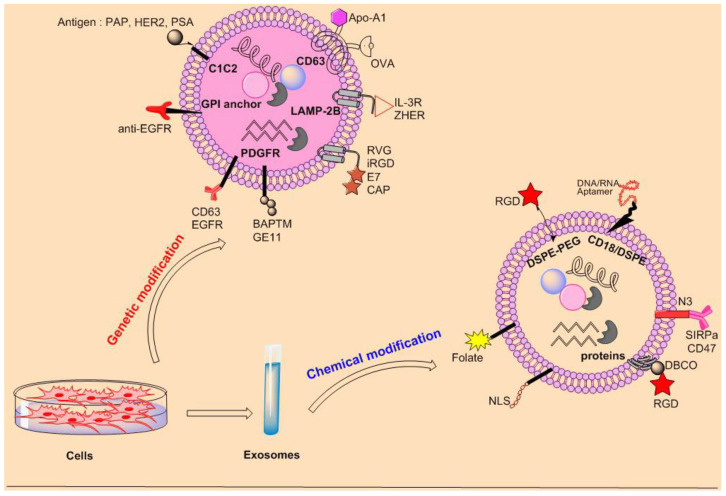
Schematic figure of the functionalization of exosomes surface. Exosomal surfaces can be functionalized by genetic modification and chemical modification. These approaches improve tumor targeting ability of exosomes.

Native exosomes/EVs have shown many advantages against synthetic nanocarriers; however, they cannot effectively accumulate in the target organs after systemic administration, particularly in targets sheltered by physiological barriers [40,65]. In recent years, scientists have been motivated to increase the targeting ability of exosomes in delivering therapeutic agents to target cells. Previous evidence shows that two main methods, genetic modification and chemical modification, are being used by researchers (Figure 4). In general, in the genetic modification strategy, exosome-producing cells are genetically modified to express biomolecules on the exosomes lumen or surface [66,67]. In the chemical modification, isolated exosomes are functionalized by chemical reactions such as click chemistry and non-covalent reactions to link therapeutic molecules to exosomes’ surface for increasing their targeting ability and for other applications such as imaging and tracking [68,69]. Exosomes produced by these ways are sometimes known as engineered exosomes, which we discuss here as the functionalization of exosomes. In the next sections, we describe and give examples of studies done in this field.

### 3.1. Genetic Modification

Genetic modification of exosomes is a useful method for the functionalization of exosomes with new properties [70,71]. In this context, exosome surface is functionalized by ligands or peptides to improve their targeting ability of them (Figure 4). In this method, as mentioned above, source cells are transfected with optional plasmids to produce exosomes containing the target protein [72]. We summarized studies that used genetic modification for drug delivery systems. Different molecules are present on exosome surfaces that can be harnessed for connecting target molecules on them. Lysosomal-associated membrane protein 2B (LAMP-2B) is present on exosomes surface, which is commonly used to fuse optional biomolecules on it. Indeed, a large N-terminal extramembrane domain of LAMP-2B is presented on the exosomes surface, providing an opportunity for researchers to connect therapeutic agents and biomolecules [73]. For example, Bellavia et al. genetically fused interleukin-3 (IL-3) to the N-terminal of LAMP-2B to improve the targeting potential of exosomes for the treatment of chronic myeloid leukemia (CML). Interleukin-3 receptor α (IL-3Rα), a receptor for IL-3, is highly expressed in CML cells. Then, they loaded these exosomes with the breakpoint cluster region (BCR)-ABL siRNA and imatinib and showed a profound anticancer effect. In vivo experiments showed that these exosomes were successfully accumulated in tumor site and suppressed cancer cells with an increase in the survival rate of xenografted mice [74]. In a similar vein, Liang and co-workers fused Her2 to the N-terminus of LAMP-2 to target colon cancer cells efficiently. The Her2-LAMP2 fusion protein was revealed to be expressed on the surface of the exosomes, which expedited the targeted cellular uptake via EGFR receptor-mediated endocytosis in tumor cells. Then, they incorporated 5-FU and miRNA-21i into these exosomes (target-Her2-LAMP2-GFP) by electroporation and incubation methods, respectively. Results showed that these exosomes accumulated in colon cancer cells and profoundly suppressed them both in vitro and xenografts BALB/c nude mice model [75]. In a study, the HEK293T cells were transduced by a lentiviral vector bearing-LAMP2b-DARPin G3 chimeric gene to produce exosomes containing DARPin G3 and then loaded them with siRNA for delivery to tumor cells, SKBR3 cells. These exosomes could specifically target SKBR3 cells and deliver siRNA molecules, inhibiting the gene expression [46]. Dendritic cells were used to express αγ integrin-specific iRGD peptide (CRGDKGPDC) on exosomes surface. The tumor-targeting ability of exosomes was improved by engineering Lamp2b fused to CRGDKGPDC. These exosomes are loaded with doxorubicin via electroporation. Doxorubicin-loaded iRGD exosomes displayed highly effective targeting ability and transfer to αv integrin-positive breast cancer cells in vitro. Intravenous injection of exosomes showed that these exosomes delivered doxorubicin specifically to tumor site, causing inhibition in tumor growth [76]. In addition, HEK293T cells were reprogrammed to concurrently express Lamp2b and KRAS siRNA in fusion with the CRGDKGPDC, iRGD peptide. Intravenous administration of iRGD-exosomes precisely could target tumor mass in vivo. The therapeutic effects were shown by the profound suppression of tumor growth in a mouse model [77]. Bai et al. prepared tLyp-1-lamp2b plasmids and transfect those into HEK293T cells. Next, the synthesized siRNA was sorted into tLyp-1 exosomes by electroporation method. In vitro experiments showed that the tLyp-1-siRNAs- exosomes had a high delivery efficacy into lung cancer and cancer stem cells [78]. Even though the efficiency of the genetic technique, the targeting peptide may be degraded in some conditions. To improve the firmness of peptides presented on the surface of the exosomes, a glycosylation motif may be linked to the N-terminus of peptide-LAMP-2B fusions [79]. Other exosomal surface proteins are the tetraspanin proteins such as CD63, CD81, and CD9, highly expressed in exosomes, which can be used to fuse with targeting molecules. For example, in mdx mice, Ran et al. fused myostatin propeptide with the second extracellular loop of CD63, which meaningfully increased its delivery efficacy and serum stability. Administration of myostatin propeptide-loaded exosomes helped muscle renovation and proliferation, causing prominent inhibition in muscle degradation and pathology [80]. ApoA-1, the key functional protein of high-density lipoprotein (HDL), is present in exosomes surface and binds to scavenger receptor class B type 1 (SR-B1) abundantly located on hepatocellular carcinoma cells. In a study, researchers genetically inserted ApoA-1 into the small extracellular loop of CD63 in 293T cells and subsequently on the surface of exosomes. Exosomes purified from ApoA-1-overexpressing donor cells were loaded with miR-26a by electroporation method. These exosomes were revealed to bind to HepG2 cells selectively via SR-B1 and at that time, captured by receptor-mediated endocytosis. The successful release of miRNA-26a in HepG2 cells reduced cell migration and proliferation [81]. Transfection with plasmid DNA encoding an OVA Ag fused to CD63 (pCD63-OVA) resulted in producing OVA-carrying exosomes. Vaccinating mice with these exosomes increased strong Ag-specific T cell responses, principally those from CD8^+^ T cells [82]. These exosomes delivered DNA vaccination meaningfully repressed tumor growth. In addition, the fluorescent protein pHluorin was fused into the small extracellular loop of CD63 to visualize the exosome secretion and uptake [83]. The transmembrane protein platelet-derived growth factor receptor (PDGFR) is also used for the functionalizing exosomes surface. Chen et al. genetically presented two types of antibodies (αCD3 UCHT1 and scFv fragments of αEGFR cetuximab) on exosome surface. In both in vitro and in vivo experiments, these exosomes were shown to encourage cross-linking of T cells and EGFR-expressing breast cancer cells and increase effective antitumor immunity [84]. Furthermore, the 37-residue glycosylphosphatidylinositol (GPI) signal peptide DAF on exosomes surface can be used to link the C-terminus of different proteins, such as a reporter protein, an antibody, or a nanobody. For example, GPI-anchored EGFR-specific nanobodies were used to target EGFR-positive human epidermoid squamous carcinoma cells [67]. In addition, exosome targeting is realized by linking the antigen to the C1C2 domain of the lactadherin protein. Treatment of mice with these exosomes results in a prominent rise in the immune response and a decrease in tumor growth [31]. In other studies, streptavidin-lactadherin and Gaussia luciferase-lactadherin were fused with lactadherin for exosome display and tracking in biological systems [85,86].

### 3.2. Chemical Modification

**Figure 5:**
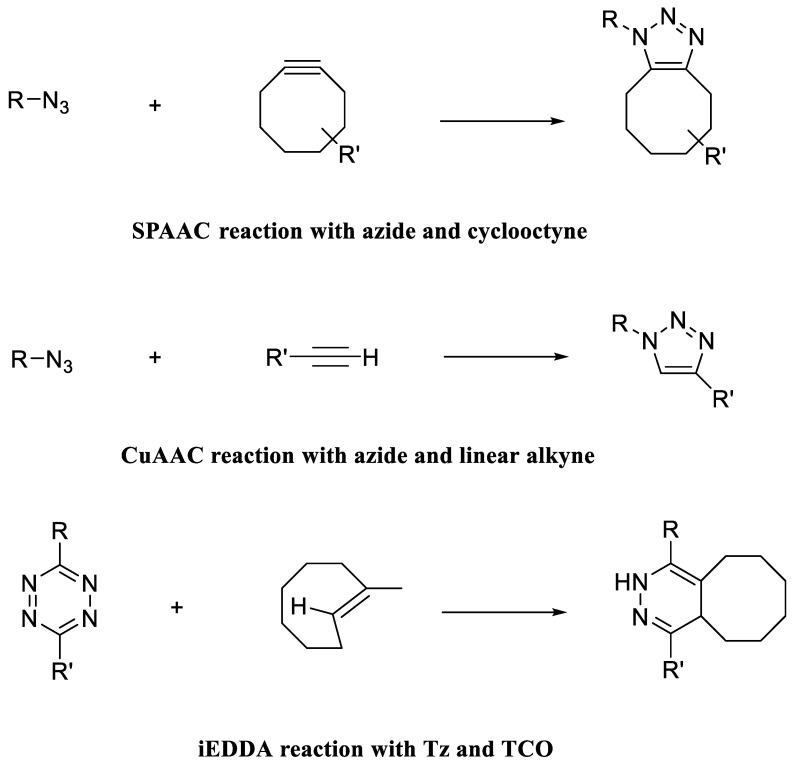
Schematics of various click chemistry reactions used to modify exosomes.

Exosomes have some interesting properties such as biocompatibility, homing ability, non-immunogenicity, blood distribution, cell specific targeting, and so on, which made them a versatile tool for drug delivery. Moreover, the chemical modifications of EVs/exosomes could not only increase their stability but also maximize their efficacy of targeting and delivery [87,88] (Figure 4). In this regard, covalent and non-covalent binding approaches have been used as two efficient chemical strategies. Non-covalent binding methods are used to load cargo on the surface of EVs/exosomes. For example, Pi et al. modified exosomes surface with cholesterol-connected RNA aptamers or folate by noncovalent binding reaction [89]. These exosomes transferred miRNA and siRNA to the tumor region with greater antitumor efficacy. Researchers used diacyllipid-aptamer as the targeting ligand to progress an aptamer-functionalized exosomes (Apt-Exos) nanoplatforms for cell type-specific delivery of molecular therapeutics. These exosomes could efficiently transfer therapeutic drugs/fluorophores to target tumor cells [88]. A membrane-targeted chimeric peptide (ChiP) was established to link a nuclear localization signal (NLS) peptide to the exosome membrane [90]. ChiP exosomes expedited the nuclear delivery of photosensitizers and improved photodynamic tumor therapy both in vitro and in vivo [90]. In case of non-covalent binding, electrostatic interactions between surface peptides and EVs played a critical role for improving exosome’s cellular uptake and cytosolic release. In one report, cationic lipid known as lipofectamine (LTX) with positive charge interacted electrostatically with the negatively charged surface membrane of a CD63-green fluorescent protein (GFP)-tagged exosome [91]. Cellular uptake investigation of prepared complex was evaluated towards HeLa and CHO-K1 cells. Results showed an increasing the cellular uptake of synthesized complex 15 and 175-fold by HeLa and CHO-K1 cells, respectively. These modifications can simultaneously improve the targeted ability of exosomes and the targeted delivery of drug-loaded exosomes. Click chemistry as an important covalent binding reaction has been widely employed to functionalize EVs. The term “click chemistry”, which was first used in its entirety by Sharpless in 2001, refers to a set of distinct and controllable reactions that must satisfy several requirements, including high yield, high specificity, simplicity, only non-toxic byproducts, and high atom economy [92]. Under physiological conditions, click chemistry reactions can take place, and the resulting chemical bonds are irreversible. As a result, click chemistry is frequently used to modify biomolecules such as proteins, lipids, and nucleic acids with different compounds. The copper (I)-catalyzed azide-alkyne 1,3-dipolar cycloaddition (CuAAC) reaction is an example of the click chemistry processes (Figure 5) that has been applied as a bioorthogonal process in the study of health sciences [93,94].

By presenting an azido moiety on the surface of the exosome through metabolic glycan synthesis and then conjugating a fluorescent dye with a strain-promoted dye, Lee and coworkers introduced a simple and efficient exosome labeling strategy. To better understand the biology and distribution of exosomes and develop exosome-based therapeutic approaches, their metabolic exosome labeling strategy may prove to be a useful tool [95]. Incorporating radioactive technetium (^99m^Tc) on the surface of the exosomes membrane is one of the alternatives to the click chemistry method. In a different fascinating study, Hwang created a straightforward technique for radiolabeling macrophage-derived exosome-mimetic nanovesicles (ENVs) with ^99m^TcHMPAO under physiological conditions, and then tracked the in vivo distribution of 99mTc-HMPAO-ENVs using SPECT/CT in living mice. Their noninvasive imaging of radiolabeled-ENVs promises a better understanding of exosome behavior in vivo for future biomedical applications [96]. The other technique uses PEG-conjugated exosomes that have been radiolabeled. It is well known that PEGylation of exosomes enhances their pharmacokinetics, permits greater tumor accumulation, and reduces their premature hepatic sequestration and clearance [96]. Recent research has shown that click chemistry can effectively be used to alter exosome-producing cells or purified exosomes to produce “tailored” vesicles.

This field is novel and several studies have been done on cancer treatment. Chemical functionalization of exosomes by click chemistry depends on the transformation of exosomal amine groups to alkynes. For example, Tian et al. by using click chemistry conjugated the peptide c(RGDyK) (Arg-Gly-Asp-D-Tyr-Lys) to the exosome surface. Then, c(RGDyK) exosomes were loaded with curcumin and injected to a transient middle cerebral artery occlusion mouse model. Results showed that these exosomes efficiently infiltrate the BBB and reduce apoptosis and inflammatory responses [97]. Similarly, in a study using click chemistry, the glioma-targeting RGE peptide (RGERPPR) was linked to exosomes by a cycloaddition reaction with sulfonyl azide. Intravenous injection of RGE exosomes caused an accumulation of exosomes in tumor regions. These glioma-targeting exosomes could be loaded with curcumin, which successfully delivered curcumin-SPION to tumor cells and induced a potent antitumor effect in mice cancer model [98]. SPION cargo of exosomes expedited magnetic flow hyperthermia therapy. By click chemistry, copper-catalyzed azide alkyne cycloaddition was used to conjugate both macromolecules and small molecules to the surface of the exosomes. This reaction showed that exosomes cross-linked with alkyne groups using carbodiimide chemistry were conjugated to a model azide, azide-fluor-545 [68]. Therefore, the exosomal amine groups can be simply functionalized with alkyne groups. Modification of exosomes surface was used to enhance immune responses against tumor cells. In this regard, Koh et al. used click chemistry to link dibenzocyclooctyne-derivatized SIRPα antibodies to Azide-modified exosomes. Tumor cells express CD47 on their surface, which can interact with SIRPα on phagocytes, resulting in phagocytosis activity of macrophages. Therefore, decorating exosomes surface with dibenzocyclooctyne-derivatized SIRPα antibodies can suppress tumor cells [99]. Therefore, these exosomes can block the CD47-SIRPα checkpoint on the surface of cancer cells. Click chemistry was used to insert amphipathic molecules into the lipid bilayer of exosomes. For instance, Kim et al. constructed and improved a formulation of paclitaxel-loaded exosomes with chemically inserted aminoethylanisamide-polyethylene glycol (AA-PEG) vector moiety to target the sigma receptor that overexpressed by lung tumor cells. The AA-PEG-vectorized exosomes enriched with paclitaxel (AA-PEG-exoPTX) displayed a high loading ability, intense capability to amass in tumor cells upon intravenous injection, and improved therapeutic effects [100]. Zhou and co-workers prepared cancer-targeting exosomes from MSCs to regulate tumor microenvironment in pancreatic ductal adenocarcinoma (PDAC) [101]. They loaded MSCs-derived exosomes with galectin-9 siRNA using an electroporation technique, and then superficially changed with oxaliplatin (OXA) as immunogenic cell death (ICD)-inducer, iEXO-OXA platform. These exosomes significantly improved tumor targeting ability and delivered the drug in the tumor region. Although click chemistry is a simple and rapid reaction method, some challenges remain. For example, this method commonly does not work specifically on a distinct exosomal amine group. In addition, non-site-specific chemical modification may guard some protein–protein interactions and change the pharmaceutics and biochemistry of the exosomes.

## 4. Future Outlooks

Exosomes may be utilized as carrier particles for several drug delivery applications. As mentioned, compared with conventional and synthetic delivery approaches, exosomes have been revealed to deliver therapeutic cargo with a low immune clearance level when injected systemically in vivo. Exosomes are flexible regarding the modification of their content and surface via different methods as well as production by various cells. For clinical translation of exosomes, there are still some problems that remain to be answered. In Box 1, we prepared the facts and gaps in exosomes biology and application. The main question is “which cells are suitable for the production of exosomes?” Several researchers used various cells for drug delivery systems. For example, animal cells (from tumor cells to stem cells), plant cells, bacteria, and body fluids are employed to load cargo. Another issue is that mass and reproducible production methods are needed to obtain continual exosomes. Although several procedures are used to obtain massive exosomes, there is no standard for this purpose and methods are relatively scattered [102,103]. In addition, exosomes isolation and characterization need technological progress to achieve pure and homogenous exosomes with a high yield efficiency [104]. Even though the therapeutic cargo can be loaded into exosomes [105], technological and reproducible handy and standard methods are required to load exosomes with the favorite drugs at a high level. The productivity of pre-loading methods is rather low, and most post-loading methods could harm the stability and integrity of exosomes [48,106]. Functionalization of exosomes is still in the beginning and it is of utmost importance that it be tested before clinical application. For improving the efficiency of the targeting ability of exosomes, further multidisciplinary research is needed to introduce a gold-standard method. Genetic modification may harm the safe nature of exosomes and chemical modification may harm exosomes’ integrity and disrupt functional molecules on the exosome surface. Progress in cancer molecular science, materials science, nanoscience, and pharmacology may overcome these problems and increase the efficacy of exosome-based cancer treatment.

Box 1 **Facts and gaps of exosomes biology and application**

Facts

- Exosomes are nanovesicles with a diameter size between 30 and 150 nm, originating from inside cells with different biological cargo.
- Exosomes are present in most body fluids and therefore can travel to different organs throughout the body.
- Exosome entity from different cells is highly variable regarding their function and cargo.
- Exosomes can encapsulate various exogenous therapeutic cargo and deliver them to target cells.
- The exosomal surface can be engineered for imaging, targeting, and therapy objects.

Gaps

- What are/is the common way/s for isolation and purification of exosomes? Exosomes are heterogeneous both in size and function, and different methods are used to isolate and purify exosomes from various sources such as cell culture medium or body fluids. Therefore, a gold-standard method is needed.
- Which source cells are suitable for the massive production of exosomes? The most important issue is large-scale good manufacturing practice (GMP)−exosome production under GMP-compliant procedures to safeguard quality, safety, and reliability. Therefore, safe and confident parental cells must be selected for exosomes yield.
- What are/is the standard/s method/s for loading exosomes with exogenous cargo?
- Which surface modification approach/es is/are most common for the improving exosomes targeting abilities? Mainly two methods, namely genetic and chemical modification approaches, are used to functionalize the surface of exosomes for increasing the tumor-targeting ability of exosomes. Exosomal surfaces are decorated with various biomolecules with a certain function. This may make it difficult for selecting a suitable and safe target residual on the surface of the exosomes.
- Do surface modification methods damage exosome structure and function? Surface modification (functionalization), especially chemical ones, may blind or harm available residuals on exosome surface. Therefore, this may affect exosomes’ pharmacokinetics and biodistribution in the body.

## 5. Conclusions

Several approaches have been used for therapeutic cargo-loading into exosomes such as pre-loading and post-loading methods. These approaches are associated with advantages and disadvantages. The inconsistency in some studies may relate to divergences in the source cells, exosomes isolation methods, and cargo properties. Large-scale production of exosomes needs GMP-compliant procedures to safeguard quality, safety, and reliability of exosomes. Therefore, for clinical application, a gold standard and universal loading method remains a challenge. In recent years, researchers have focused on the surface functionalization of exosomes for increasing their tumor-targeting ability. In this regard, isolated exosomes are genetically and/or chemically modified to link some agents such as ligands, peptides, aptamers, and antibodies. It seems that click chemistry has quickly appeared as a widespread and main method for the functionalization of exosomes chemically. For exosome modification, it is essential to fully comprehend the structure and cargo of the exosomes membrane at the target site, and choose the suitable targeting ligands. In addition, for the effective progress of clinically appropriate exosome-based therapy for cancer, forming approaches for the precise measurement of therapeutic cargo/drugs in exosomes would be required to discover a therapeutically operative concentration of exosomes. Thus, approaches for the measureable and qualitative observing of exosomes and exosome-loaded drugs to the tumor microenvironment should be advanced. Despite a robust attempt at explaining different aspects of exosomes—such as the biogenesis pathway, interactions with target cells, loading methods, and exosomal surface modification—there is still much scrutinizing to be done around incorporating the multidimensional abilities of exosomes into the drug delivery system.

## Figures and Tables

**Table 1:** Advantages and limitations of exosomes loading methods.

Active Methods	Examples	Advantages	Limitations	Ref.
physical induction	electroporation	High efficacy and is more efficient than chemical transfection	Depends on cargo types May affect the Zeta potential and colloid stability of exosomes Induce aggregation of siRNAs	[46,47]
	sonication	Compared with co-incubation and electroporation, sonication produces the highest loading efficiency	Damage the EVs structure Must be performed in an ice bath	[48,49]
	freeze-thaw cycle	Rapid and easy loading	Low loading efficiency, increase the size of EVs, induces aggregation	[50,51]
	extrusion	Compared with co-incubation, extrusion can produce homogeneous EVs	change the Zeta potential and membrane proteins in EVs	[52,53,54]
Chemical induction				
	saponins	High loading efficiency	Potential toxicity; cause hemolysis Need extra purification phase	[55,56]
	liposomes	High loading efficiency, quickly and easily load	Potential toxicity	[57,58]
	calcium chloride	High loading efficiency simple and stable	Potential toxicity	[59]

## Data Availability

Not applicable.
